# Pathological characteristics and HER2 status of gastric cancer in Chinese high-incidence area

**DOI:** 10.1097/MD.0000000000047517

**Published:** 2026-02-13

**Authors:** Huijuan Wang, Jinxia Gao, Xingbing Li, Zhichuan Chen, Liuxue Ma, Wenqing Yan, Peng Zhang, Xiaoyun Wu

**Affiliations:** a Liangzhou Hospital, Wuwei, Gansu Province, China; b WuWei Center for Disease Control and Prevention, Wuwei, Gansu Province, China; c WuWei Hospital of Traditional Chinese Medicine, Wuwei, Gansu Province, China.

**Keywords:** HER2 positive rate, high incidence gastric cancer areas, pathological characteristics

## Abstract

A retrospective analysis was conducted on the positive rate of HER2 in gastric cancer in the Chinese high-incidence area of Wuwei from 2015 to 2024. The pathological characteristics of HER2-positive gastric cancer were explored to provide a basis for precise diagnosis and treatment in high-incidence areas of gastric cancer. A retrospective study design was adopted to collect and analysis the HER2 expression status of gastric cancer surgical specimens from 2015 to 2024. A total of 416 gastric cancer surgical specimens were collected for HER2 detection, and the positive rate of HER2 was 9.13% (38/416), including 33 males (9.68%) and 5 females (6.67%), with no statistical significance (*P* >.05). There were 24 (8.63%) HER2 positive patients over 60 years old, 11 (10.67%) HER2 positive patients between 50 and 59 years old, and 3 (8.6%) HER2 positive patients under 50 years old, with no statistical significance (*P* >.05). In cTNM stages, HER2 was positive in 7 patients (5.65%) in stage Ⅰ, 16 patients (10.32%) in stage Ⅱ, 14 patients (10.61%) in stage Ⅲ, and 1 patient (20%) in stage Ⅳ, with no statistical difference between groups (*P* >.05). The positive rate of tubular adenocarcinoma HER2 in WHO classification was 10.5%. Other tissue types were 0%, there were differences (*P* <.05); The positive rate of HER2 in well-differentiated adenocarcinoma was 10.84%, in moderately differentiated adenocarcinoma 16.44%, and in poorly differentiated adenocarcinoma 8.25%, with a difference between moderately and poorly differentiated adenocarcinoma (*P* <.05). The positive rate of HER2 in T3 infiltration depth was 15.97% higher than that in T1/T2/T4 (*P* <.05). The positive rate of HER2 in cardiac/fundus was 16.26% higher than that in other sites (*P* <.05). The proportion of patients with intestinal stype gastric cancer was the largest in Lauren classification (59.13%), but the HER2 positive rate of mixed type was significantly higher than that of other Lauren classification (*P* <.001). Patients with lymph node metastasis (13%) were significantly higher than those without lymph node metastasis (4.66%), and the difference was statistically significant (*P* <.01). The research data indicate that correlation between the expression of HER2 in areas with a high incidence of gastric cancer and the pathological characteristics.

## 1. Introduction

Gastric cancer is one of the common malignant tumors of the digestive system. Its incidence and mortality rates vary across different regions. The latest data from the International Agency for Research on Cancer shows^[1]^ that in 2022, there were nearly 20 million new cases of tumors worldwide, among which 96,840 were new cases of gastric cancer, accounting for 4.9% of all new tumors; 659,800 deaths occurred, accounting for 6.8% of all deaths. Simultaneous data from the Chinese Cancer Center show that,^[2]^ in 2022 there were 4.8247 million new cases of tumors in China, with 358,700 new cases of gastric cancer, ranking fifth among new tumor cases, and 260,400 deaths, ranking third in the tumor mortality spectrum. Currently, the disease burden of gastric cancer in China is severe and shows a high incidence and mortality level.^[3]^ Wuwei City, Gansu Province, as a high-incidence area of gastric cancer, still has gastric cancer incidence and mortality at the top of the tumor disease spectrum in this province.^[3,4]^ Currently, targeted and immunotherapy breakthroughs have successfully been applied to the comprehensive treatment of gastric cancer, among which human epidermal growth factor receptor (EGFR) 2 (HER2), as an important target of gastric cancer, targeted therapy drugs have achieved significant clinical results.^[5,6]^ Analyzing the expression of HER2 in high-incidence areas of gastric cancer over 10 years and its correlation with pathological features has important guiding significance for achieving individualized, precise treatment and prognosis judgment for gastric cancer patients in this region.

## 2. Materials and methods

### 2.1. Data sources

The data used in this study were collected from Liangzhou Hospital in Wuwei City. The research subjects were surgical patients diagnosed with gastric cancer through pathology from 2015 to 2024, and a total of 416 cases were included. Clinical data of the patients, including age, gender, tumor location, tumor differentiation degree, Lauren classification, invasion depth, clinical stage, and lymph node metastasis, were collected. Inclusion criteria for cases: Patients with gastric cancer who underwent the first surgery; All underwent HER2 immunohistochemical staining and/or fluorescence in situ hybridization (FISH) examination. This research has been approved by the Human Ethics Committee of Liangzhou Hospital of Wuwei City.

### 2.2. Detection methods

**Figure 1.**
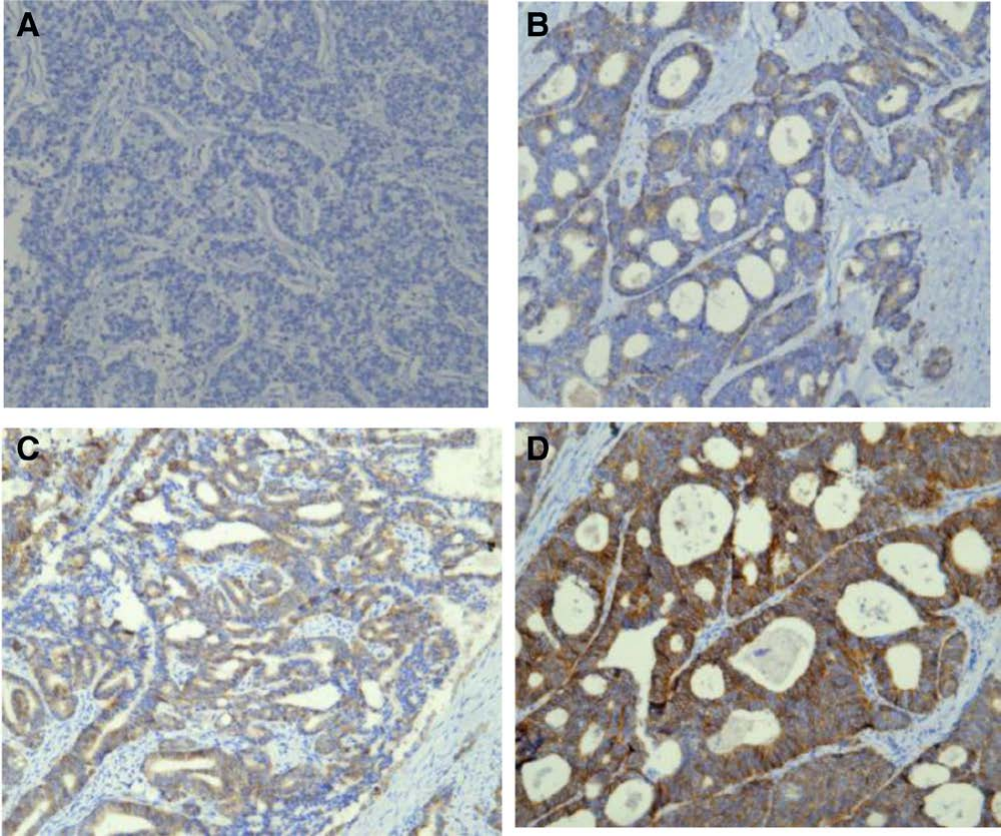
Expression of HER2 protein in the tested samples detected by IHC. 1A: IHC 0+ (×100). 1B: IHC 1+ (×100). 1C: IHC 2+ (×100). 1D: IHC 3+ (×100). HER2 = human epidermal growth factor receptor 2, IHC = immunohistochemistry.

**Figure 2.**
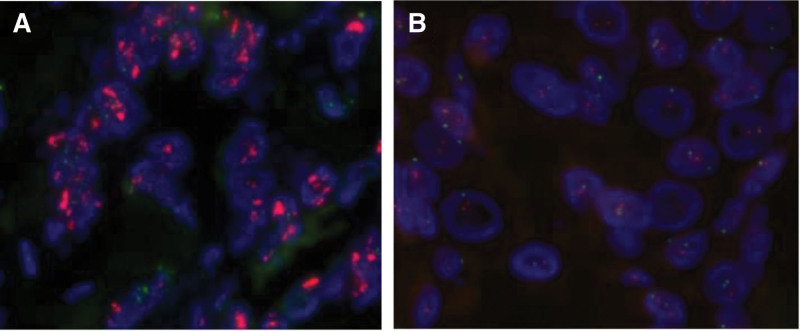
Expression of HER2 protein in the tested samples detected by fluorescence in situ hybridization (FISH). (A) HER2 FISH+ (×1000). (B) FISH- (×1000). FISH = fluorescence in situ hybridization, HER2 = human epidermal growth factor receptor 2.

The expression of HER2 in tumor tissues was detected using immunohistochemistry (IHC) and FISH methods. IHC is the preferred method for detecting HER2 in gastric cancer. The IHC test results are classified as 0, 1+, 2+, and 3 + (Fig. 1), where IHC 3 + is determined as HER2 positive, IHC 1 + and IHC 0 are determined as negative; IHC 2 + is “uncertain” and requires further FISH testing. The FISH test result with amplification is determined as HER2 positive (Fig. 2).

### 2.3. Statistical analysis

All the cases included in the study were registered using EpiDate 3.02 software. Double entry by 2 persons was conducted. The HER2 positive status of gastric cancer was analyzed. SPSS26.0 software was used to conduct statistical analysis on the collected data. The count data were expressed as percentages (%). The χ^2^ test was used for comparison between groups, and a difference was considered statistically significant when *P* <.05.

## 3. Result

### 3.1. Overall HER2 expression rate

**Figure 3.**
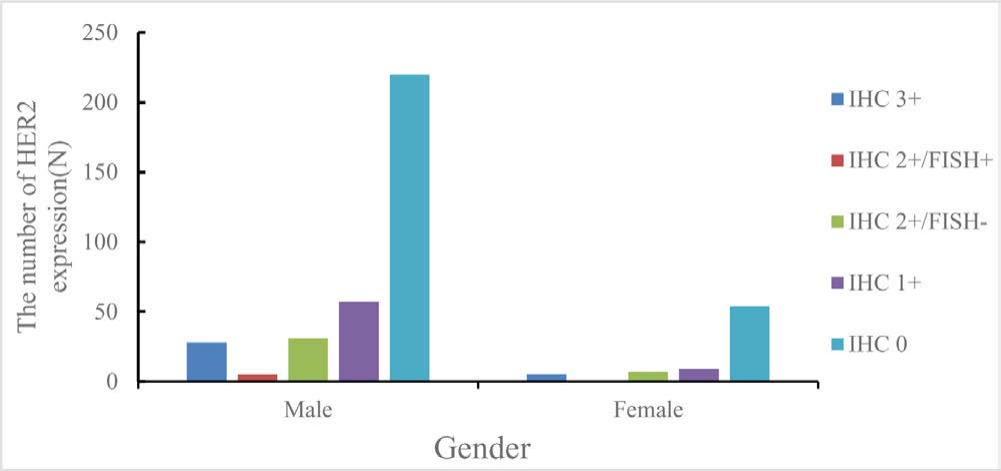
The number of HER2 expression. HER2 = human epidermal growth factor receptor 2.

This study retrospectively collected the HER2 test results of 416 gastric cancer patients, including 341 males (accounting for 81.97%) and 75 females (accounting for 18.03%). The summary of HER2 test results from 2015 to 2024 (Table 1 and Fig. 3) shows that the HER2 positive rate was 9.13% (38/416). Among them, 33 patients had IHC 3 + (7.93%), including 28 males and 5 females; 43 patients had IHC 2 + (10.34%), including 36 males and 5 females. For the IHC 2 + patients, FISH examination was performed, and 5 cases were FISH positive, all of whom were male patients, with a positive rate of 11.63%. There were 66 patients with IHC 1 + and 274 patients with IHC 0.

**Table 1:** The number of HER2 expression.

Gender	Male	Female	Total
IHC 3+	28	5	33
IHC 2+/FISH+	5	0	5
IHC 2+/FISH-	31	7	38
IHC 1+	57	9	66
IHC 0	220	54	274
Total	341	75	416

FISH = fluorescence in situ hybridization, HER2 = human epidermal growth factor receptor 2, IHC = immunohistochemistry.

### 3.2. The relationship between HER2 expression in gastric cancer and pathological characteristics

The expression of HER2 in gastric cancer is correlated with clinical pathological characteristics (Table 2). The analysis results showed that the positive rate of HER2 in adenocarcinoma was higher than that in other tissue types (*P *<.05); the positive rate of HER2 in well-differentiated adenocarcinoma was 10.84%, in moderately differentiated adenocarcinoma was 16.44%, and in poorly differentiated adenocarcinoma was 8.25%. There was a difference in the positive rate of HER2 between moderately differentiated and poorly differentiated adenocarcinoma (*P* <.05), while there was no statistical significance in the positive rate of HER2 among well-differentiated, moderately differentiated, and poorly differentiated adenocarcinoma (*P *>.05). The proportion of intestinal-type gastric cancer patients was relatively large at 59.13% (246/416). There was a significant statistical difference in the positive rate of HER2 among different Lauren subtypes (*P *<.001). Among them, the positive rate of HER2 was the highest in the mixed type at 16.13% (5/31), followed by the intestinal type at 12.60% (31/246) and the diffuse type at 1.44% (2/139). The positive rate of HER2 in T3 infiltration depth was 15.97% (23/167), which was significantly higher than that in T1/T2/T4 (*P* <.05); the positive rate of HER2 in the tumor site of the cardia/gastric fundus was 16.26% (20/123), which was significantly higher than that in the gastric body/gastric antrum/gastric pylorus sites at 6.14% (18/293), and the difference was statistically significant (*P *<.05); the positive rate of HER2 in patients with lymph node metastasis was 13% (29/223), which was significantly higher than that in patients without lymph node metastasis at 4.66% (9/193), and the difference was statistically significant (*P* <.01). There was no statistical difference in cTNM stage, age, and gender with HER2 expression (*P *>.05).

**Table 2:** Correlation between HER2 status and clinicopathological features.

Clinicopathological features	N	HER2 status		χ^2^	*P*-value
		Negative (%)	Positive (%)		
Age of diagnosis (year)					
<50	35	32 (91.43)	3 (8.6)	0.394	.821
50–59	103	92 (89.32)	11 (10.67)		
≥60	278	254 (91.37)	24 (8.63)		
Gender					
Male	341	308 (90.32)	33 (9.67)	0.671	.413
Female	75	70 (93.33)	5 (6.67)		
WHO classification					
Tubular adenocarcinoma	362	324 (89.50)	38 (10.50)	5.038	.025
Other*	54	54 (100)	0 (0)		
WHO differentiation†					
Well	83	74 (89.16)	9 (10.84)	3.858	.154
moderately	73	61 (83.56)	12 (16.44)		
Poorly	206	189 (91.75)	17 (8.25)		
Lauren classification					
Intestinal	246	215 (87.40)	31 (12.60)	15.308	.00
Mixed	31	26 (83.87)	5 (16.13)		
Diffue	139	137 (98.56)	2 (1.44)		
Depth of infiltration					
T1	59	57 (96.61)	2 (3.39)	7.880	.049
T2	162	151 (93.21)	11 (6.79)		
T3	167	144 (86.23)	23 (15.97)		
T4	28	26 (92.86)	2 (7.14)		
Location of tumor					
Cardia/fundus	123	103 (83.74)	20 (16.26)	11.85	.01
Body/antrum/pylorus	293	275 (93.86)	18 (6.14)		
Lymph node metastasis					
Yes	223	194 (87.00)	29 (13.00)	8.673	.003
No	193	184 (95.34)	9 (4.66)		
cTNM stage					
I	124	117 (94.35)	7 (5.65)	3.879	.248
II	155	139 (89.68)	16 (10.32)		
III	132	118 (89.39)	14 (10.61)		
IV	5	4 (80)	1 (20)		

HER2 = human epidermal growth factor receptor 2, TNM = tumor node metastasis, WHO = World Health Organization.

* Includes sig-ring cell carcinoma, adeno-squamous cell carcinoma, mucinous carcinoma and other tissue types.

† Analyze the differentiation of tubular adenocarcinoma.

## 4. Discussion

HER2 is a tyrosine kinase receptor and a member of the EGFR family. The HER2 gene is located on human chromosome 17 (17q21). HER2 mainly inhibits apoptosis by activating the PI3K/Akt signaling pathway, enhances the invasive and migratory abilities of tumor cells, and promotes cell cycle progression, accelerates cell proliferation and differentiation by activating the Ras/MAPK signaling pathway.^[7–12]^ Overexpression or amplification of HER2 can lead to the formation of homodimers or heterodimers with other members of the EGFR family (such as HER3), thereby continuously activating downstream signaling pathways and driving tumor progression.^[7]^ Overexpression of HER2 has been confirmed in various malignant solid tumors, such as breast cancer and lung cancer.^[13,14]^ The overexpression of HER2 in gastric cancer was first reported in 1986.^[15]^ With the breakthroughs in targeted and immunotherapy, gastric cancer has entered the era of precision treatment. The success of the ToGA study has provided a new direction for treatment.^[6]^ A multi-center, real-world study in China (EVIDENCE)^[16]^ confirmed that Chinese patients with HER2-positive gastric cancer who received trastuzumab treatment had significantly longer median OS and PFS compared to those who did not receive it. Trastuzumab combined with chemotherapy has become the standard first-line treatment for HER2-positive advanced gastric cancer. Studies have shown that in HER2-positive gastric cancer, the abnormal activation of the PI3K signaling pathway (such as PIK3CA mutations) is one of the important mechanisms for trastuzumab resistance; preclinical experiments have demonstrated that the combination of PI3K inhibitors (such as copanlisib) with anti-HER2 therapies (such as trastuzumab or lapatinib) can synergistically inhibit tumor cell proliferation and overcome drug resistance.^[8]^ Genomic analysis revealed that HER2 alterations encompass not only gene amplification but also base substitutions, which may affect the efficacy of targeted drugs, highlighting the importance of comprehensive molecular classification.^[17,18]^ For instance, the classification based on operable genes divides HER2-positive gastric cancers into separate clusters, and such patients are more likely to benefit from anti-HER2 treatment.^[18]^ In the context of the tumor microenvironment, overexpression of HER2 may affect immune cell infiltration (such as macrophage polarization and T cell function), thereby participating in the process of immune escape. It has been reported that HER2 activation mutations can induce the autocrine signaling pathway of transforming growth factor β1 (TGF-β1), and enhance its expression through the Rac1 and JNK pathways, thereby altering the immune regulatory state of the tumor microenvironment.^[19]^ In the tumor microenvironment driven by HER2, the infiltration of regulatory T cells (Treg),^[20]^ myeloid-derived suppressor cells,^[21]^ and tumor-associated macrophages^[22]^ has been observed to increase. These immunosuppressive cells may jointly promote immune escape. The above research mechanisms provide a theoretical basis for the combined application of HER2-targeted therapy and immunotherapy.

A global multi-center study has shown^[23]^ that the positive rate of HER2 in gastric cancer is approximately 10.4% to 20.2%. The positive rate of HER2 in European and American countries is slightly higher than that in Asian countries. The positive rate of HER2 in gastric cancer in China is 12% to 13%.^[24]^ This study shows that the HER2 positive rate in the high-incidence area of gastric cancer was 9.13% (38/418), which was slightly lower than the levels reported previously both globally and domestically, and higher than the 8.8% result of the domestic study on HER2 expression in gastric cancer.^[25]^ This might be due to the fact that Wuwei City is a region with low medical resources, and the FISH technology was developed relatively late. 43 samples of IHC2 + paraffin were all reexamined by FISH from 2015 to 2024. This might be caused by the fact that the paraffin samples were stored at room temperature for a long time or the previous tissue fixation time was too long (>48 hours), resulting in some false-negative results. At the same time, there is a high heterogeneity in HER2 expression in gastric cancer.^[26–28]^ Without standardizing the screening of HER2 heterogeneity expression, it might also be a reason for the false-negative results. If we calculate based on the average positive rate of FISH in IHC2 + being 25%,^[25]^ the potential positive rate of HER2 in this region is 10.8%, which is the same as the reported positive rate worldwide.

The expression of HER2 is of vital importance in the development of precise detection and personalized treatment for gastric cancer. Both domestic and international guidelines recommend that all cases of gastric adenocarcinoma confirmed by pathological diagnosis should undergo HER2 testing.^[24]^ In this study, HER2 testing was conducted on all surgical gastric cancer specimens of various tissue types. The results showed a statistically significant difference (*P* <.05). The HER2 positive rate in gastric adenocarcinoma was significantly higher than that in other tissue types, such as signet ring cell carcinoma, adeno-squamous carcinoma, and mucinous carcinoma, etc. Further analysis of the correlation between HER2 expression and pathological tissues revealed that the positive rate of HER2 in the cardia/gastric fundus was higher than that in the gastric body/gastric antrum/ pylorus. This suggests that gastric cancers of different tissue types and locations may have different biological behaviors and molecular characteristics, providing a direction for the precise diagnosis and treatment of gastric cancer. From the Luaren classification perspective, the positive rate of HER2 in the intestinal type was significantly higher than that in the diffuse type and the mixed type, which is consistent with the previous results of the ToGA screening study.^[29]^ In terms of the degree of adenocarcinoma differentiation at WHO, the positive rate of HER2 in moderately differentiated adenocarcinoma was the highest, followed by well-differentiated and poorly differentiated adenocarcinoma. This indicates that the abnormal expression of HER2 may be related to the occurrence process of gastric cancer. In this study, it also means that moderately differentiated adenocarcinoma is more likely to benefit from targeted therapy with HER2. As the depth of tumor invasion increases and lymph node metastasis occurs, the positive rate of HER2 rises, indicating that HER2 overexpression is associated with the invasiveness and metastatic ability of gastric cancer.^[30]^ The results of this study show that the HER2 positive rate is the highest in stage T3. Since the gastric cancer specimens included in the study mainly fall within the T2/T3 stages, the reason for the relatively low HER2 positive rate in stage T4 might be that patients in the T2/T3 stage have a better overall condition and tumor situation, making them more suitable for surgical resection. However, for patients in stage T4, due to the deep and extensive tumor infiltration, involving surrounding vital organs and blood vessels, the surgical difficulty is extremely high and the risks are too high. Therefore, they mostly choose conservative treatments such as radiotherapy, chemotherapy, and targeted therapy, resulting in a relatively small number of surgical specimens included in the T4 group and a relatively lower HER2 positive rate detected.

Wuwei City, Gansu Province, is located at the eastern end of the Hexi Corridor. It is one of the areas with a high incidence of gastric cancer in China.^[31–33]^ Although the incidence and mortality of gastric cancer across the country have been decreasing in recent years, the incidence and mortality of gastric cancer in Wuwei City still rank first in the city’s tumor disease spectrum.^[4]^ The high-risk group for gastric cancer in Wuwei City consists of male patients over the age of 50. Particularly, there are 278 cases of patients over 60 years old. In this study, the proportion of patients over 50 years old in the gastric cancer specimens included was 91.6% (381/416), among which 82.0% (341/416) were male, which is consistent with previous reports.^[4]^ However, there was no statistical significance in the expression of HER2 with age and gender. The reasons for the high incidence of gastric cancer in Wuwei City may be related to local diet, environment, helicobacter pylori infection and other factors.^[4]^ Although certain achievements have been made in the prevention and control of gastric cancer in Wuwei City, the precise diagnosis and treatment of gastric cancer still face severe challenges.

This study was a single-center study that only included surgical specimens from Wuwei region. Although it could reflect some local characteristics, the sample size (418 cases) was relatively limited, and the inclusion of late-stage cases (such as T4 stage) due to surgical indications was insufficient, which may lead to limitations in the results. Although the HER2 detection process combined with IHC and FISH was followed, some paraffin samples showed false negatives due to long-term storage at room temperature and nonstandard tissue fixation (>48 hours). At the same time, the heterogeneity of HER2 expression was not standardizedly investigated (such as multi-region biopsy and detection in different lesion sites), which to some extent reduced the true positive rate of HER2. This study did not systematically correlate the subsequent treatment plans (such as trastuzumab combined with chemotherapy) and long-term survival prognosis of the patients, and there were difficulties in evaluating the predictive value of HER2 status for treatment efficacy and survival.

In conclusion, Wuwei City is one of the areas with a high incidence of gastric cancer. By thoroughly analyzing the correlation between HER2 expression in gastric cancer patients and their pathological characteristics, it can effectively guide the precise targeted treatment of gastric cancer in clinical practice. The research shows that the HER2 positive rate in this region is consistent with the global reports, and the pathological characteristics are in line with the previous reports. In the future, it is necessary to conduct multi-center collaboration in conjunction with the areas along the Hexi Corridor and other regions with a high incidence of gastric cancer, to increase the sample size and balance the proportions of each TNM stage, and to clarify the regional differences and actual levels of HER2 positivity in these high-incidence areas. Introducing high-throughput sequencing and digital PCR molecular detection technologies, we have established a standardized evaluation process for HER2 expression heterogeneity (such as spatial transcriptomics and multi-region biopsy analysis), reducing false negatives and analyzing the impact of heterogeneity on treatment response. At the same time, we integrate genomic, transcriptomic, and proteomic data to analyze the co-expression patterns of HER2 with PD-L1, Claudin 18.2, FGFR2b, etc, and explore “HER2 and other targets” dual-targeted treatment strategies. We also establish a long-term follow-up model to clarify the predictive value of HER2 status for patient prognosis. Taking into account the high-salt diet and Helicobacter pylori infection in the Wuwei area, we will conduct studies on epigenetics (such as DNA methylation and miRNA regulation) to analyze the regulatory mechanism of environmental factors on HER2 expression. This will provide new directions for the primary prevention and mechanism research of gastric cancer in high-incidence areas.

## Acknowledgments

Huijuan Wang is the first author of this manuscript. Huijuan Wang acquired and analyzed the data, drafted and revised the manuscript. The data was also acquired by Jinxia Gao, Xingbing Li, Liuxue Ma, Zhichuan Chen, Peng Zhang. The data was also analyzed by Wenqing Yan, Jinxia Gao. The conception, design, and final approval of the submitted version were performed by Xiaoyun Wu. Conception and design were also done by Huijuan Wang.

## Author contributions

**Data curation:** Jinxia Gao.

**Formal analysis:** Jinxia Gao.

**Investigation:** Xingbing Li.

**Resources:** Zhichuan Chen, Liuxue Ma, Wenqing Yan, Peng Zhang.

**Software:** Peng Zhang.

**Writing – original draft:** Huijuan Wang, Xiaoyun Wu.

**Writing – review & editing:** Huijuan Wang.
